# Application of Xanthan Gum as a Pre-Treatment and Sharpness Evaluation for Inkjet Printing on Polyester

**DOI:** 10.3390/polym11091504

**Published:** 2019-09-16

**Authors:** Hongmei Cao, Li Ai, Zhenming Yang, Yawei Zhu

**Affiliations:** 1 College of Textile and Clothing Engineering, Soochow University, 199 Renai Road, Suzhou 215123, China; 2 Changzhou Vocational Institute of Textile and Garment, ChangZhou Key Laboratory of Eco-Textile Technology, Changzhou 213164, China

**Keywords:** xanthan gum, pre-treatment, inkjet printing, polyester fabric, sharpness

## Abstract

Inkjet printing on polyester fabric displays versatile environmental advantages. One of the significant benefits of inkjet printing is a dramatic enhancement of the printing quality. In this study, xanthan gum—a bio-based thickening agent accompanied by several salts—was adopted for the pretreatment of polyester fabric aiming at improving the sharpness and color depth of inkjet printed patterns. The influences of four metal salts (NaCl, KCl, CaCl_2_ and MgCl_2_) on inkjet printing performance were studied. More importantly, a quantitative method for evaluating the sharpness of an inkjet printed pattern was established according to the characteristics of anisotropy and isotropy of diffusion and adsorption of ink droplets on a fiber surface. Results showed that xanthan gum along with a low dosage of bivalent salts can significantly improve the color depth (K/S value) and sharpness of the printed polyester fabrics. It is feasible to evaluate the sharpness of inkjet printed polyester fabrics using a five-stage system, selecting the inkjet ellipse coefficient (T) and inkjet ellipse area (S), which can provide a quantitative and rapid evaluation method for defining inkjet printing.

## 1. Introduction

Inkjet printing technology has been widely applied in the textile field due to its ability to do short runs of production and lower cost advantages, when compared to conventional dyeing and printing processes [1]. Inkjet printing is becoming more important and more popular for polyester fiber printing. As a surface modification technique, the pretreatment of polyethylene terephthalate (PET) fibers for inkjet printing is very important. It increases the color intensity, enlarges the color gamut, improves the color fastness, and also plays an important role in the determination of the printing accuracy of inkjet printing [2,3]. In general, PET fibers for inkjet printing are surface modified with atmospheric-pressure air/He plasma [4] and a commercial pretreatment agent [2]. By considering the environment protection issues, the eco-friendly natural materials such as xanthan gum [5], chitosan or chitosan derivative [6], and β-cyclodextrin (β-CD) [7] are widely applied for the surface modification of PET fibers prior to inkjet printing. With application of these pretreatment agents, great improvements have been achieved in inkjet printing on PET fibers. However, the accuracy of printing is still not to the desired quality level. The pretreatment agents have a great influence on the diffusion shape and printing accuracy [8] by affecting the ink droplet spreading behavior and the trajectory of droplets [9,10].

Xanthan gum is a microbial polysaccharide branched structure consisting of a (1–4)-linked-β-d glucose backbone with ionic side chains. Low concentration of xanthan solution can display a thixotropic flow behavior [11,12]. Xanthan gum has been widely used in medicine, food technology, chemical industry, textile and other fields [13,14,15,16], due to its desirable thickening, suspension, thermal resistance, acid and alkali resistance, electrolyte resistance and other properties. In the field of textile dyeing and finishing, xanthan gum is mainly used in printing, dyeing and sewage treatment [17,18]. When it is used as a printing paste, xanthan gum easily generates weak gel phenomenon [19], due to its unique double helix structure and strong intermolecular interaction. However, xanthan gum has not been applied to the pretreatment of PET fibers prior to inkjet printing to date and its feasibility requires exploration.

Xanthan gum is a relatively cheap renewable natural resource. Previous studies reported that hydrophobic interactions like hydrogen bonds abundantly exist in xanthan gum and surfactant aqueous systems (such as anionic surfactant-SDS or nonionic surfactant-Tween 80) [20]. The rheological properties of xanthan gum are changed due to mixed starches [21], metal divalent ions (calcium, magnesium and iron ions) [22] and sodium alginate [23]. Currently, there is no report about the pretreatment of xanthan gum on inkjet printing of PET fibers.

In this study, salt-containing xanthan gums are applied to the pretreatment of PET fibers prior to the inkjet printing. This method improves the color performance of disperse dye inkjet as the environmentally friendly pretreatment agent on PET fibers, and establishes a quick evaluation of the sharpness of the printed polyester fabrics, using the adsorption and diffusion of the ink drops on fibers treated with salt-containing xanthan gum. It is a method which is used to quickly identify the effect of pretreatment agents for disperse dye inkjet printing and it is also helpful to prevent the blocking of inkjet printing heads, when developing the dye ink product.

Outline sharpness of inkjet printing is very important. The method used to evaluate the sharpness of inkjet printing depends on line width of printed patterns [9,24,25], but it is difficult and inconvenient to evaluate the sharpness of inkjet printing. When the ink drops reach the fabric surface, the adsorption and diffusion of the ink drops are anisotropic due to the different meridional and zonal surface structure of fabric. Moreover, the ink drops are elliptical, which directly affects the sharpness of the inkjet printing. When the surface structure of the fabric is isotropic in the warp and weft directions, the ink droplet is a circular shape on the surface of fabric. Therefore, it is convenient to evaluate the sharpness of the printed polyester fabrics according to the characteristics of the anisotropy and isotropy of the adsorption and diffusion of ink droplets on the fabric surface. A new method for evaluating the sharpness of inkjet printing is established, which can enrich the characterization method for evaluating the sharpness of inkjet printing by ink drop experiment.

## 2. Materials and Methods

### 2.1. Materials

A scoured PET fabric was supplied by Jiangsu Lan-Gan Group (Jiangsu, China). The fabric was plain weave using 17.8 tex warp count and 4.4 tex weft count yarns. The weight of the fabric was 73.2 g/m^2^ with density 827 ends/10 cm in the warp direction and 358 ends/10 cm in the weft direction. Salt (NaCl, KCl, CaCl_2_, MgCl_2_, chemical pure), sodium hydroxide (chemical pure), sodium hyposulfite (chemical pure) and Detergent LS (C_25_H_46_NNaO_5_S, anion surfactant, industrial pure) were purchased from market. Xanthan gum (food level) was supplied by Ordos Zhongxuan biochemical Co. LTD (Ordos, China). Disperse red ink (D2551) was supplied by DuPont Co. (Wilmington, DE, USA).

### 2.2. Preparation of Salt-Containing Xanthan Gum

Xanthan gum paste was prepared with a mass ratio of 0.3%. It was stirred and dispersed using 80 °C hot water at 700–900 r/min for 1h. Four kinds of salts (NaCl, KCl, CaCl_2_ and MgCl_2_) with different concentrations were added into xanthan gum paste, respectively. The mixture was stirred for 30 min, and the mixture was placed overnight before use.

### 2.3. Printing of Polyester Fabric

**Figure 1:**
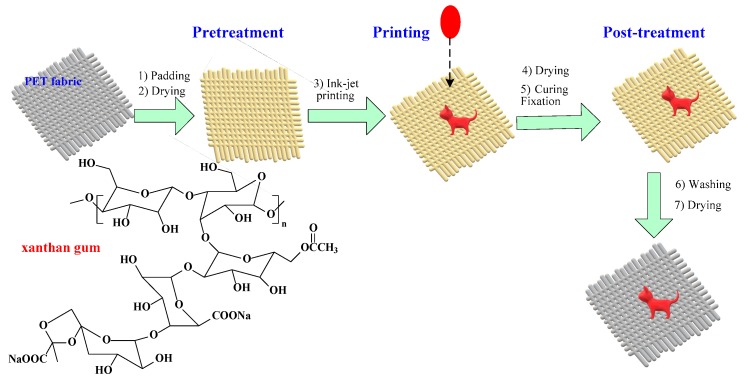
Flowchart of printing process on the pretreated polyethylene terephthalate (PET) fabric.

Figure 1 shows the preparation process of saline xanthan gum and inkjet printing of PET fabric and post-treatment. Firstly, PET fabric was padded (hydro-extraction effect was 80%) and dried with different salt-containing xanthan gum solutions with a continuous setting and curing machine (M-TENTER, Rabbit Co., Taiwan, China), and was then dried at 110 °C for 3 min. Printing was implemented using an inkjet printer (Stylus Photo R330, Epson, Nagano, Japan) at the resolution of 720 × 720 dpi with a commercial disperse red inkjet ink (D2551). Then, the PET fabric was dried at 110 °C for 3 min and put through a curing fixation process at 190 °C for 1 min. The cured PET fabric was finally washed in a washing liquid containing 1 g/L NaOH, 1 g/L sodium hyposulfite and 1 g/L Detergent LS with a liquor ratio of 1:30 at 75 °C for 5 min.

K/S value measurements of the printed PET fabric were obtained using a spectrophotometer (Ultra Scan XE, Hunter-Lab., Reston, VA, USA). The spectrophotometer was set to exclude specular reflection with a large aperture (D_65_ and 10° observer). K/S values with different wavelengths ranging from 400 to 700 nm within the visible spectrum and measured at 10 nm intervals were calculated according to Equation (1), then summed up as the K/S average value after four tests. The higher the K/S value, the higher the dye-uptake will be, resulting in better color yield.

K/S = (1 − R)^2^/2R (1)

where K is equal to the absorption coefficient; S is equal to the scattering coefficient; and R is equal to the reflectance of the colored sample.

The rubbing fastness test was performed according to the standard (ISO 105-X12:2016) using Model 670 type friction fastness machine (James H and Heal Co., Halifax, West Yorkshire, England). The washing fastness test of the printed fabric was performed according to the standard (ISO 105-C02:2013) using wash fastness tester (Roaches International Co., Leek, Staffordshire, England). The light fastness test was performed according to the standard (AATCC TM16-2004) using ATLAS CT 3000+ Weather-Ometer / Fade-Ometer (Atlas Material Testing Technology, Chicago, IL, USA). The light source was Xenon-Arc exposure for 40 h. The air permeability test was performed according to the standard (ISO 9237:1995) using a YG461 air permeability tester (Nantong Hongda experiment instruments co. LTD, Jiangsu, China). The test area of the sample was 20 cm^2^, and air pressure was kept at 100 Pa.

FTIR (Fourier transform infrared spectroscopy) tests were obtained from Nicolet 5700 (FTIR; Nicolet 5700, Thermo Electron Co., Waltham, MA, USA). The frequency range was 650–4000 cm^−1^ by KBr pellets technique and a resolution of 4 cm^−1^. SEM (scanning electron microscopy) tests were obtained from S-4800 (SEM, S-4800, Hitachi, Tokyo, Japan). The magnification of the SEM is 1000 times.

Outline sharpness of the printing samples were examined using a 3D ultra-depth of field microscope (Vhx-5000, Keyence Co., Osaka, Japan). The line widths of the printed lines in both warp and weft directions were specified as 1.5 pounds. The practical widths were photographed by 3D ultra-depth of field microscope and Pinnalce Studio Version 8.0 Software (Keyence Co., Osaka, Japan) with a magnification of 100 multiples. The line widths of the collected images are measured at 20 points in the warp and weft directions, respectively, and the average of the line widths is calculated. 

### 2.4. Ink Drop Experiment and New Methods for the Definition of Inkjet Printing

**Figure 2:**
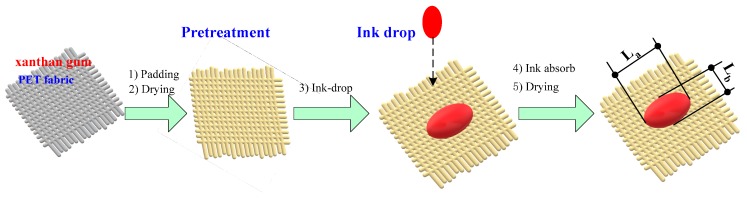
Flowchart of ink drop experiment on the pretreated PET fabric.

Figure 2 shows the preparation process of the saline xanthan gum and ink drop experiment. First, PET fabric was treated with a saline xanthan gum solution. Then, refer to the testing method of water absorbency of textiles (JIS L 1907-2010, Japanese Industrial Standard). The fabric was suspended, the four corners of the textile stretched to ensure that the fabric is kept in the stretched tile state. By using home-made micro-separator, the ink (10 μL) drops one centimeter vertically over the fabric. After the ink had completely absorbed for 30 min, the PET fabric was dried at 110 °C for 3 min. Finally, the length of the long axis (L_a_, cm) and the short axis (L_b_, cm) forming the ink mark ellipse are determined.

According to the characteristics of anisotropy and isotropy of the adsorption and diffusion of ink droplets on the fabric surface, the sharpness of inkjet printing can be characterized by the length of the long axis, the length of the short axis and the area of the inkblot ellipse formed after the ink was completely absorbed.

After the ink was completely absorbed by the fabric, the formation of the ink ellipse coefficient (T) and the area of the ink ellipse (S, cm^2^) were measured by the length of the long axis (L_a_, cm) and the short axis (L_b_, cm).

The new outline sharpness of the inkjet ink pattern samples were evaluated by the inkblot ellipse coefficient (T) and inkblot ellipse area (S).

The calculation is shown in Equations (2) and (3). The data was tested 20 times and the results are averaged.

T = L_b_/L_a_ (2)

(3) $$S=\frac{\pi }{4}\cdot L_{a}\cdot L_{b}.$$

L_a_ was the long axis of the inkblot ellipse (it was typically in the weft direction of the fabric), cm.
L_b_ was the short axis of the inkblot ellipse (it was typically in the warp direction of the fabric), cm.

When the surface properties of the fabric in the warp and weft directions are isotropic, the closer the blot ellipse coefficient (T) is to 1.0, the better the definition of droplet adsorption and diffusion. The sharpness of droplet adsorption diffusion was evaluated according to the inkblot ellipse coefficient (T) and inkblot ellipse area (S). The evaluation criteria consist of five grades, with Grade 1 being the lowest and Grade 5 being the highest.

Grade 1, S_p_ ≥ S_u_, the definition rating is the poorest.

Grade 2, S_p_ < S_u_, 0 < T ≤ 0.70, poor clarity rating.

Grade 3, S_p_ < S_u_, 0.70 < T ≤ 0.80, medium clarity rating.

Grade 4, S_p_ < S_u_, 0.80 < T ≤ 0.95, good definition rating.

Grade 5, S_p_ < S_u_, 0.95 < T≤ 1.0, excellent definition rating.

S_p_ was the area of the pretreated fabric droplet (cm^2^), S_u_ was the area of the untreated fabric droplet (cm^2^).

## 3. Results and Discussion

### 3.1. Effect of Salt Concentration on the K/S Value of Inkjet Printing

**Figure 3:**
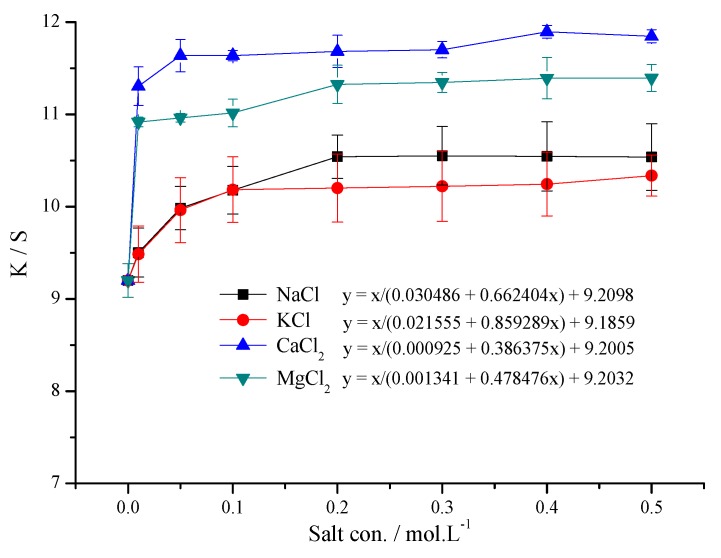
Effect of salt concentration on the K/S value of inkjet printing.

Figure 3 shows the K/S value of the jet ink printed fabric treated with four kinds of saline xanthan gum. Salt concentrations were varied in the range of 0–0.5 mol/L.

The impact of bivalent salts (Ca^2+^ and Mg^2+^) on the K/S value of inkjet printed fabric was significantly larger than that of monovalent salts (Na^+^ and K^+^). With the increase of salt concentrations, the K/S value of inkjet printed fabric increases. Table 1 shows a relationship the K/S value (y) and salt concentration (x/mol.L^-1^). The K/S value has a relationship with salt concentration (x/mol.L^-1^), y = x/(C_1_ + C_2_∙x) + C_0_. The variance test shows that the decision coefficient (R^2^) is close to 1, the F value is bigger, and the *p*-value is very small. It shows that the matched curve of mathematical mode is reliable. When the concentration of salt ranged from 0.01 to 0.2 mol/L, the K/S value showed a significant increase compared with salt-free xanthan gum solutions. When the salt concentration continued to increase from 0.2 to 0.5 mol/L, C_1_ was small, and C_1_ + C_2_∙x ≈ C_2_∙x, the change of K/S value of salt was small. As the K/S value decreases, the order of the cation salts is Ca^2+^ > Mg^2+^ > Na^+^ > K^+^. It could be that XG solution behaved like a pseudo-plasticity fluid and the relationship between its viscosity and shear rate could be described by the power-law model. The concentration of XG solution had little influence on the linear viscoelastic range [26]. The apparent viscosity and viscoelasticity of XG solutions decrease with the addition of inorganic cations [27]. Xanthan’s viscoelastic and morphological properties may be tuned by addition of surfactants and salt [28]. Compared with the unsalted XG, NaCl and CaCl_2_ tended to produce a more drastic decrease of apparent viscosities of XG dispersions than KCl and MgCl_2_ [29]. Chloride and formate potassium brines not only increase the viscosity of the solution but also extend the shear thinning behavior to temperatures near 200 °C, however, the ordered conformation still dominates the rheological behavior [30]. The water absorption of XG/WCS (water chestnut starch) solution was improved drastically by the addition of NaCl [31]. It illustrates that xanthan gum with bivalent salt, which is easy to absorb and permeates with low viscosity, is more suitable for the pretreatment of inkjet printing. In addition, the salt has the effect of reducing the potential of the fiber surface and the double layer, which can help to reduce the electrostatic repulsion of the dye with the fabric surface (xanthan gum), promotes the absorption and fixation of the dye with the fibers, and also improves the dry rubbing fastness grade and apparent color gain of the dye.

Under the optimal pretreatment condition, the K/S of treated PET fabric using 0.1 mol/L CaCl_2_ and 0.3% xanthan gum was higher than that of treated PET fabric only using 0.3% xanthan gum, it can be calculated that K/S increased by 26.99%. The color fastness grade of treated PET fabric using CaCl_2_ and xanthan gum was 4–5 (dry rubbing), 4 (wet rubbing), 4–5 (washing), 5 (light), respectively. The higher dry rubbing fastness grade was achieved with treated PET fabric using CaCl_2_ than that using only xanthan gum (dry rubbing fastness grade was 4). Other color fastness grade was the same as using CaCl_2_ and xanthan gum, using only xanthan gum, respectively.

**Figure 4:**
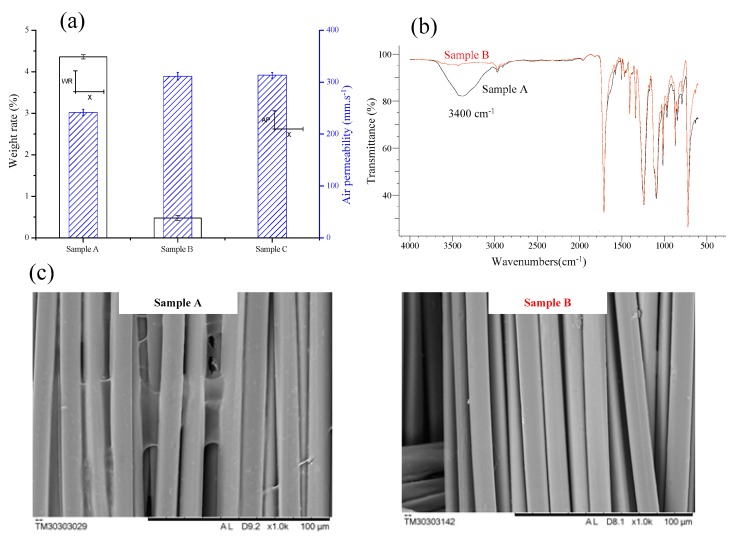
Effect of xanthan gum of treated PET fibers. (**a**) Change of weight ratio and air permeability of fibers. (**b**) Fourier transform infrared spectroscopy (FTIR) spectrum of fibers. (**c**) Scanning electron microscopy (SEM) image of fibers (×1000). Sample A was PET fibers treated using CaCl_2_ and xanthan gum. Sample B was PET fibers treated by post-treatment. Sample C was untreated PET fibers.

Figure 4 shows the weight ratio and air permeability (Figure 4a), FTIR spectrum (Figure 4b) and SEM image (Figure 4c) of printed fabric treated with the xanthan gum pretreatment and finally washing.

When PET fibers were prepared using CaCl_2_ and xanthan gum, the weight ratio of the adsorbed xanthan gum and air permeability of fibers was 4.36%, and 241.7 mm/s, respectively (Figure 4a). Figure 4b,c clearly show that sample A has the characteristic peak at 3400 cm^−1^, which is assigned to the O–H stretching of xanthan gum, and the polymer film of xanthan gum is adhesive on the treated fiber surfaces. When PET fibers were applied in the post-treatment, the weight ratio and air permeability of fibers were 0.48%, and 311.6 mm/s, respectively. It was indicated that water soluble xanthan gum is easily washed away from the fabric during the post-treatment washing process. Xanthan gum did not affect the microstructural characterization of PET fibers, due to the water solubility and ease of washing. In fact, the amounts of xanthan gum were washed away 88.96% during post-treatment. The air permeability of fibers was almost the same as untreated PET fibers (air permeability was 313.3 mm/s).

Xanthan gum is shown to be minimally retained when observed by SEM images of the fibers, i.e. the relatively smooth surfaces which are visualized on sample B. The sample B characteristic peak of O–H stretching of xanthan gum disappears. Therefore, it does not have an adverse influence on PET fibers, which retain the porous nature of the fabric after post-treatment.

### 3.2. Effect of Salt Concentration on the Outline Sharpness of Inkjet Printing

**Figure 5:**
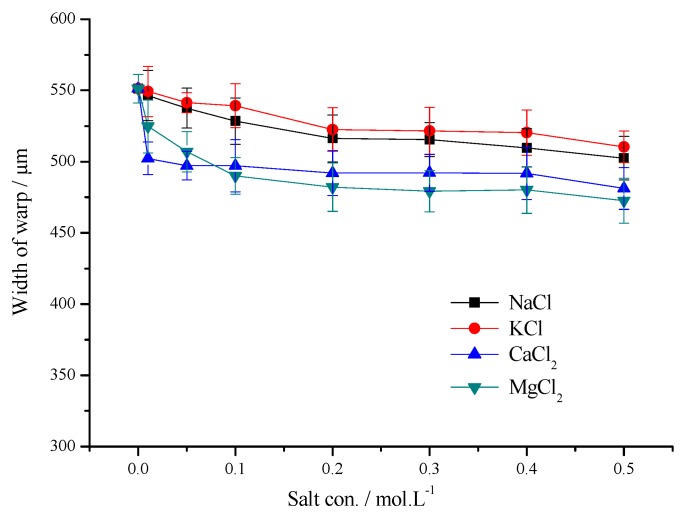
Effect of salt concentration on the width of warp printing direction.

**Figure 6:**
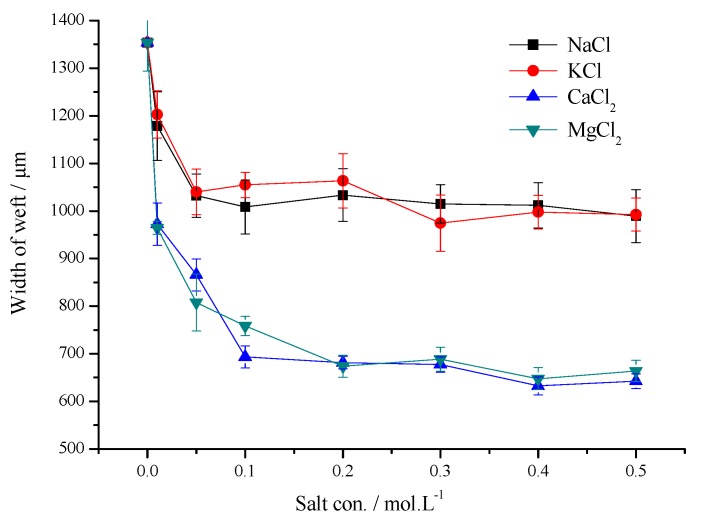
Effect of salt concentration on the width of weft printing direction.

Figure 5 and Figure 6 show the printed lines width in the warp and weft directions on the pretreatment with four kinds of saline xanthan gum. Salt concentration was 0–0.5 mol/L, respectively.

The printed lines in the warp direction were narrower than those in the weft direction. Due to the difference between fabric structure and capillary effect, the ink droplet infiltration along the latitudinal direction is more serious, and the width of the latitudinal ink droplet decreases more obviously under the action of salt, but the meridional direction still has better printing sharpness than the latitudinal direction.

The printed lines on the pretreated fiber using bivalent salt were narrower than those using monovalent salt both in the warp and weft directions. When the salt concentration is low (0.01–0.1 mol/L), the line width in warp direction improved 8.89–9.83% (CaCl_2_), 4.81–11.12% (MgCl_2_), the line width in the weft direction improved 28.17–48.78% (CaCl_2_), 28.75–43.97% (MgCl_2_), respectively. When the salt concentration continues to increase, the line width in the warp direction improved more distinctly than those in the weft direction using monovalent salts. However the line width in the weft direction improved more distinctly than those in the warp direction using bivalent salts. When the salt concentration is high (0.4–0.5 mol/L), the line width in the warp and weft directions improved a small amount. It could be that when PET fibers were treated using the saline XG solution, the salt leant the fabric surface a certain positive charge, which slowed down the seepage of ink drops caused by electrostatic repulsion among xanthan gum, fabric and dye, and improved the fiber surface’s adhesion to the ink. The salt ions reduce the surface tension of the fabric, which makes it difficult for the ink droplets to spread on the fiber surface, and the area of the formed ink droplets is significantly less than that of the untreated fabric [20,32]. In the process of inkjet printing, the ink droplets are ejected from the nozzle, and the area formed by the ink droplets on the fabric lessens due to the effect of salt, which is conducive to the infiltration of the ink droplets into the fiber, and slowing down the infiltration of the ink droplets along the warp and weft directions, which results in decreased sharpness of the printing pattern [8,25,33].

### 3.3. New Evaluation Method of Outline Sharpness for Inkjet Printing

In general, outline sharpness and the surface appearance of inkjet printing fabrics are very important, however it is difficult to quantify the outline sharpness with data. There has not been any studies about the outline sharpness, besides the line width of actual inkjet printing.

Table 2 shows the results of the ink drop experiment and line width of actual inkjet printing. As shown in Table 2, there is a significant difference in the adsorption and diffusion of ink drops in the warp and weft directions when the ink drops are directly dropped on the surface of the fabric. We clarify the definition of inkjet printing for five grades. 

Grade 1, the definition rating of the fiber is the poorest. L_a_ and L_b_ are 2.5, and 1.5 cm, respectively. The inkblot ellipse area (S) is 2.94 cm^2^ (>2.86 cm^2^, untreated fiber). Line widths of actual printing in the warp and weft directions are 1353.3, and 551.3 μm, respectively. The definition rating of the treated-fiber is also the poorest, the definition of the inkjet printing is Grade 1. 

Grade 2, the definition rating of the treated fiber is due to the poor clarity rating. Compared to Grade 1, the L_a_, L_b_ and inkblot ellipse area are decreased, and the line widths of the actual printing in the warp and weft directions are also decreased. The range of L_a_, L_b_, T (the blot ellipse coefficient) and S are 1.9–2.4 cm, 1.2–1.5 cm, 0.57–0.70, and 1.79–2.71 cm^2^, respectively. The range of line widths of actual printing in the warp and weft directions are 964.2–1202.5, and 502.3–549.3 μm, respectively. The average values of L_a_, L_b_, and S are 2.18 ± 0.12 cm, 1.36 ± 0.08 cm, and 2.34 ± 0.22 cm^2^, respectively. The average value of line widths of actual printing in the warp and weft directions are 1044.77 ± 53.27, and 528.80 ± 11.67 μm, respectively.

Grade 3, the definition rating of the treated fiber is applied for the medium clarity rating. The range of L_a_, L_b_, T and S are 1.7–1.8 cm, 1.3–1.4 cm, 0.72–0.78, and 1.73–1.98 cm^2^, respectively. The range of line widths of actual printing in the warp and weft directions are 989.2–1012.0, and 502.4–520.4 μm, respectively. 

Grade 4, the definition rating of the treated fiber is considered the good definition rating. The range of L_a_, L_b_, T and S are 1.3–1.5 cm, 1.2–1.4 cm, 0.92–0.93, and 1.22–1.65 cm^2^, respectively. The range of line widths of actual printing in the warp and weft directions are 807.6–992.5, and 507.3–510.3 μm, respectively. 

Grade 5, the definition rating of the treated fiber is the excellent definition rating. The range of L_a_, L_b_, T and S are 0.4–1.2 cm, 0.4–1.2 cm, 1.00, and 0.13–1.13 cm^2^, respectively. The range of line widths of actual printing in the warp and weft directions are 632.4–758.3, and 472.6–497.1 μm, respectively. The average values of L_a_, L_b_, and S are 0.59 ± 0.27 cm, 0.59 ± 0.27 cm, 0.35 ± 0.31 cm^2^, respectively. The average values of line widths of actual printing in the warp and weft directions are 675.77 ± 23.87, and 485.84 ± 6.78 μm, respectively.

The length of the ink drops and line width of actual printing in the weft direction are large, while the length of the ink drops and line width of actual printing in the warp direction are short. The fabric presented has obvious anisotropy in warp and weft directions, which mainly affects the orientation and organizational structure of fibers [34]; the ink was elliptical and the T value was low. The salt, especially the bivalent salt, can significantly reduce the length of the ink blots in the warp and weft directions of the fabric. The ink area decreased, the ink shape changed to a circular shape, and the T value tended to 1.0. When the bivalent salt concentration is 0.1–0.5 mol/L, the warp and weft of fabric are isotropic, the lengths of warp and weft ink are equal, and the T value is 1.0.

Therefore, the "short axis/long axis" value of ink droplets forming ink blots is introduced as an index to characterize the definition of inkjet printing ink droplets, which can quantify the influence of pretreatment on the definition of inkjet printing. By considering the difference of ink absorption and diffusion between pretreated and untreated fabric surface properties, the index of ink absorption and diffusion area was introduced. When the S of pretreated cloth is larger than untreated cloth, it is impossible to improve the sharpness of inkjet printing. And the S of pretreated cloth smaller than untreated cloth is a sufficient condition to improve the sharpness of inkjet printing.

Table 3 shows that the clarity of drop is intrinsically related to the sharpness of the inkjet printing. this can be observed clearly as the "long/short axis shaft" value can reflect the latitude and longitude inkjet printing to the size of anisotropy, and also conforms to the observer the definition estimation results of the visual design style, thus establishing that the drops of clarity rating method is feasible. This provides a new method for quantitative evaluation of inkjet printing resolution.

## 4. Conclusions

The pre-treatment of polyester fabric with xanthan gum and salt can improve the color depth and printing clarity of inkjet printing, and the divalent salt can obtain higher K/S values and printing clarity. The optimal pretreatment condition was 0.3% xanthan gum paste and 0.1 mol/L calcium chloride. K/S increased by 26.99% compared with those using only 0.3% xanthan gum paste. 

Pretreatment on fabric with xanthan gum and salt can significantly affect the fabric surface diffusion and adsorption performance, which affects the fabric and fiber surface of both isotropic and anisotropic surfaces. Using the five level system, the method of ink elliptical coefficient (T) and the ink ellipse area (S) to evaluate the pretreatment of polyester fabric inkjet printing sharpness is feasible. The evaluation method is as follows:

Grade 1, S_p_ ≥ S_u_, the definition rating was the poorest,

Grade 2, S_p_ < S_u_, 0 < T ≤ 0.70, poor clarity rating,

Grade 3, S_p_ < S_u_, 0.70 < T ≤ 0.80, the clarity rating is medium,

Grade 4, S_p_ < S_u_, 0.80 < T ≤ 0.95, the clarity rating is good,

Grade 5, S_p_ < S_u_, 0.95 < T ≤ 1.0, the clarity rating is excellent.

## Figures and Tables

**Table 1:** Effect of saline xanthan gum on the K/S value.

Salt	Mathematical Mode	Variance Test		
		R^2^	F-value	*p*-value
NaCl	y = x/(0.030486 + 0.662404x) + 9.2098	0.9896	238.6056	0.0000
KCl	y = x/(0.021555 + 0.859289x) + 9.1859	0.9909	271.7458	0.0000
CaCl_2_	y = x/(0.000925 + 0.386375x) + 9.2005	0.9943	432.5051	0.0000
MgCl_2_	y = x/(0.001341 + 0.478476x) + 9.2032	0.9633	65.6802	0.0003

**Table 2:** Relation of ink drop experiment and line width of actual printing.

Definition of Grades	Ink Drop Experiment				Line Width of Actual Printing		
	L_a_ (cm)	L_b_ (cm)	T	S (cm^2^)	Warp (μm)	Weft (μm)	Observe
Grade 1	2.5	1.5	0.60	2.94	551.3	1353.3	○
Grade 2	2.3	1.3	0.57	2.35	546.4	1178.5	◎
	2.4	1.4	0.58	2.64	549.3	1202.5	◎
	2.4	1.4	0.58	2.64	539.3	1054.6	◎
	2.2	1.3	0.59	2.25	522.6	1063.1	◎
	2.2	1.3	0.59	2.25	516.3	1033.2	◎
	2.1	1.3	0.62	2.14	521.6	974.2	◎
	1.9	1.2	0.63	1.79	502.3	972.1	◎
	2.2	1.4	0.64	2.42	537.6	1032.0	◎
	2.3	1.5	0.65	2.71	541.4	1040.0	◎
	2.0	1.3	0.65	2.04	524.8	964.2	◎
	2.2	1.5	0.68	2.59	528.5	1008.2	◎
	2.0	1.4	0.70	2.20	515.5	1014.6	◎
Grade 3	1.8	1.3	0.72	1.84	509.6	1012.0	●
	1.7	1.3	0.76	1.73	502.4	989.2	●
	1.8	1.4	0.78	1.98	520.4	997.4	●
Grade 4	1.3	1.2	0.92	1.22	507.0	807.6	☆
	1.3	1.2	0.92	1.22	497.2	865.7	☆
	1.5	1.4	0.93	1.65	510.3	992.5	☆
Grade 5	1.2	1.2	1.00	1.13	490.0	758.3	★
	1.1	1.1	1.00	0.95	497.1	693.2	★
	0.8	0.8	1.00	0.50	482.1	673.6	★
	0.4	0.4	1.00	0.13	492.0	681.3	★
	0.4	0.4	1.00	0.13	492.2	677.1	★
	0.4	0.4	1.00	0.13	491.8	632.4	★
	0.4	0.4	1.00	0.13	481.2	642.3	★
	0.4	0.4	1.00	0.13	479.3	688.3	★
	0.4	0.4	1.00	0.13	480.1	647.4	★
	0.4	0.4	1.00	0.13	472.6	663.8	★

(1) Observe definition: ○ (worst clarity rating ), ◎ (poor clarity rating),● (medium clarity rating), ☆ (good definition rating), ★ (excellent definition rating). (2) Ink drop experiment of untreated fiber: L_a_ = 2.6 cm, L_b_ = 1.4 cm, S = 2.86 cm^2^, T = 0.54.

**Table 3:** Results of the ink drop experiment, grade of definition and pattern of actual printing.

Definition of Grades	T	Pattern I	Pattern II	Definition of Marginal	Definition of Internal
Grade 1	0.60	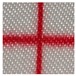	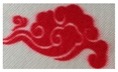	○	○
Grade 2	0.57	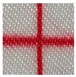	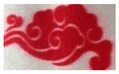	◎	◎
Grade 2	0.61	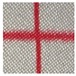	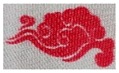	◎	◎
Grade 3	0.72	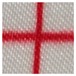	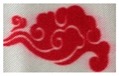	●	●
Grade 3	0.78	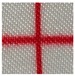	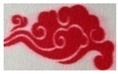	●	●
Grade 4	0.92	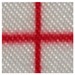	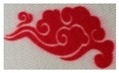	☆	☆
Grade 4	0.93	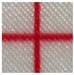	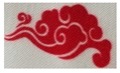	☆	☆
Grade 5	1.00	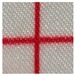	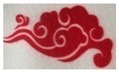	★	★

Pattern I, 30 mm × 30 mm, 1.5 pound, 720 dpi × 720 dpi. Pattern II, 30 mm × 15 mm, 720 dpi × 720 dpi.
